# Association of genetically instrumented HMGCR inhibition with the therapeutic role of prostate cancer: a Mendelian randomization study and supporting *in vitro* experiments

**DOI:** 10.3389/fphar.2025.1701869

**Published:** 2026-01-12

**Authors:** Xiaojie Hao, Jingjun Mu

**Affiliations:** Department of Urology, Shanxi Province Cancer Hospital, Taiyuan, China

**Keywords:** genetically instrumented, HMGCR inhibition, *in vitro* experiments, Mendelian randomization, prostate cancer

## Abstract

**Background:**

The role of 3-hydroxy-3-methylglutaryl-coenzyme A reductase (HMGCR), which is one of the primary targets of statin drugs, in prostate cancer (PCa) remains controversial. This study aimed to investigate the causal relationship of genetically instrumented HMGCR inhibition with the therapeutic role of PCa through Mendelian randomization (MR) and validate the findings by cell experiments.

**Methods:**

We selected single nucleotide polymorphisms (SNPs) associated with statins use by targeting HMGCR. We used three genome-wide association studies (GWASs) datasets for PCa: PCa (GWAS ID:ieu-b-85, Sample size = 140,254), PCa (GWAS ID:ieu-b-4809, Sample size = 182,625), PCa (GWAS ID:ebi-a-GCST006085, Sample size = 140,254). The inverse variance weighted (IVW) method served as the primary MR approach to validate the suitability of the selected SNPs and to explore the causal relationship between statins use and PCa. To assess the robustness of the MR findings, a comprehensive sensitivity analysis, steiger directionality tests and colocalization analyses were performed. Finally, we validated the MR findings by treating two PCa cell lines with Atorvastatin.

**Results:**

We ultimately selected 7 SNPs associated with HMGCR. The IVW results indicated that HMGCR inhibition decreased the risk of PCa. Per 1 standard deviation (SD, mg/dL) decrease in low-density lipoprotein cholesterol (LDL-C) was associated with a 16.7% reduction in PCa risk: PCa (GWAS ID: ieu-b-85, Sample size = 140,254) (odds ratio (OR) = 0.833; 95% confidence interval (CI) = 0.726–0.954; P-adjusted = 0.014); Per 1 SD (mg/dL) decrease in LDL-C was associated with a 1.5% reduction in PCa risk: PCa (GWAS ID: ieu-b-4809, Sample size = 182,625) (OR = 0.985; 95% CI = 0.973–0.998; P-adjusted = 0.023) and 16.2% reduction in PCa risk: PCa (GWAS ID: ebi-a-GCST006085, Sample size = 140,254) (OR = 0.838; 95% CI = 0.734–0.956; P-adjusted = 0.014). Results of sensitivity analysis showed MR finding was robust. However, our colocalization results indicated that no shared genetic variants were found in the HMGCR region. Results of cell experiments also demonstrated that statins use could promote apoptosis of PCa cells.

**Conclusion:**

HMGCR inhibition reduces the risk of PCa, and this protective effect is independent of its lipid-lowering action. Our findings provide strong genetic support for initiating RCTs to investigate the therapeutic potential of statins for partial PCa patients.

## Introduction

Prostate cancer (PCa) is a malignancy with high global incidence and mortality rates: According to Global Cancer Statistics 2022, there were approximately 1,466,680 new cases of PCa worldwide in 2022, ranking it the fourth most common cancer overall, while the number of deaths reached 396,792, making it the eighth leading cause of cancer-related mortality (Bray et al., 2024; Schafer et al., 2025). Among male cancer patients, PCa ranks second in incidence and fifth in mortality across all cancer types (Bray et al., 2024; Schafer et al., 2025). The rising incidence and mortality of PCa impose a substantial health and economic burden, particularly in regions such as Africa, Asia, and Latin America (Van Blarigan et al., 2025; Siegel et al., 2023). In addition to expanding PCa screening for early diagnosis, exploring effective prevention and treatment strategies represents a critical approach to alleviating the disease burden associated with PCa (Seibert et al., 2023; Plym et al., 2023; Fong et al., 2023).

Statins primarily exert their lipid-lowering effects by inhibiting 3-hydroxy-3-methylglutaryl-coenzyme A reductase (HMGCR), thereby reducing hepatic cholesterol biosynthesis (Lincoff et al., 2024; Zeng et al., 2025; Paparodis et al., 2024). They are clinically widely used for the treatment of hyperlipidemia and coronary heart disease (Lincoff et al., 2024; Zeng et al., 2025; Paparodis et al., 2024). However, a growing body of research suggests that statins may also possess potential anti-tumor effects: For instance, studies by Murtola et al. have indicated that statins may delay or inhibit the progression of PCa (Murtola et al., 2017; Joshua et al., 2022; Roy et al., 2024; Roy et al., 2025), while other research, including studies by Amiri et al., have found no significant association between statins use and PCa risk (Amiri et al., 2025; Chavarriaga et al., 2025). Current investigations into the effects of HMGCR inhibition on PCa remain predominantly reliant on retrospective studies, and their conclusions are subject to ongoing debate. Due to ethical constraints, conducting randomized controlled trials (RCTs) remains challenging.

MR is extensively used to evaluate potential causal relationships between exposures and outcomes (Chen et al., 2025). Unlike conventional observational studies, MR mitigates reverse causation by leveraging genetic variants as IVs that are randomly allocated at conception, thereby reducing confounding biases (Wang et al., 2025; Zhou et al., 2025). Moreover, MR analyses circumvent ethical limitations (Zhang et al., 2025). This study employs MR to explore the effect of HMGCR inhibition on PCa and validates the results through *in vitro* cell experiments.

## Materials and methods

### Study design

**FIGURE 1:**
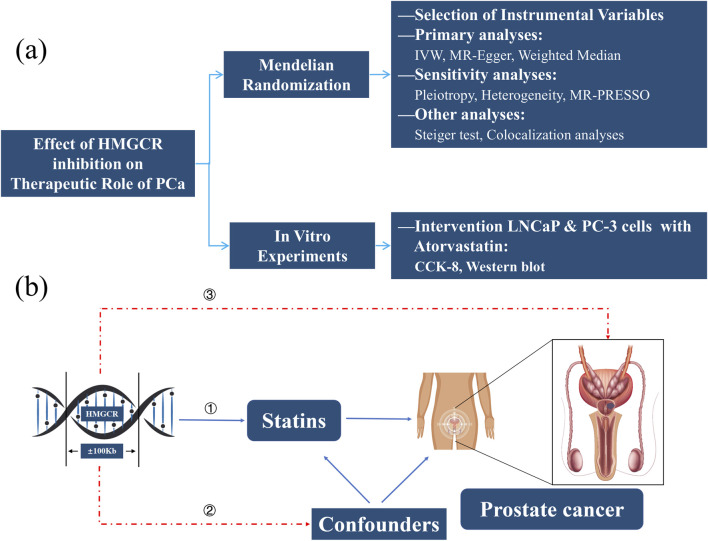
Study Design Flowchart **(a)** and Assumptions of MR **(b)**. HMGCR, 3-hydroxy-3-methylglutaryl-coenzyme A reductase; PCa, Prostate cancer; IVW, Inverse-variance weighted method; MR, Mendelian randomization; CCK-8, Cell counting kit-8.

Figure 1a presented the study design flowchart of this research: We first selected SNPs associated with HMGCR as instrumental variables (IVs), and then conducted MR to explore the therapeutic effect of HMGCR inhibition on PCa. Sensitivity analysis, Steiger directionality tests, and Colocalization analysis were performed to enhance the robustness of the MR findings. In addition, we validated the results *in vitro* by treating two types of human PCa (LNCaP and PC-3) with Atorvastatin and conducting CCK-8 assays and Western blot experiments.

Figure 1b presented the key assumptions of MR. MR studies must satisfy three key assumptions: (1) the IVs must be robustly associated with the exposure (HMGCR); (2) the selected IVs must be independent of confounders; and (3) the IVs must influence the outcome solely through the exposure pathway, without direct or alternative causal pathways.

### Outcomes sources

We obtained all free genome-wide association studies (GWASs) data from the IEU Open GWAS project (https://gwas.mrcieu.ac.uk/).

We obtained GWAS datasets for Low-density lipoprotein (LDL) (Sample size = 173,082) from Global Lipids Genetics Consortium (GLGC); To enhance the reliability of the results, we selected two GWAS datasets for coronary heart disease (CHD) and three for PCa, respectively (Supplementary Table S1).

### Selection of IVs

Four criteria were utilized for selecting suitable SNPs (Huang et al., 2021): ① We identified variants within a 100 kb window of the HMGCR gene that were significantly associated with LDL levels (p < 5.0 × 10^−8^) and the location of the HMGCR gene on the chromosome can be found on the National Center for Biotechnology Information (NCBI) (https://www.ncbi.nlm.nih.gov/); ② We ensured low linkage disequilibrium among selected SNPs (r^2^ < 0.3) to maximize instrument strength; ③ We retained only common SNPs, and minor allele frequency (MAF) >1% was considered as common SNP; ④ We excluded weak IVs according to F-statistic (F = β^2^/SE^2^) and F > 10 was considered as strong IV (Perry et al., 2021; Zhu et al., 2022). We applied to same criteria to select SNPs associated with Proprotein convertase subtilisin/kexin type 9 (PCSK9) and Niemann-Pick C1-Like 1 (NPC1L1), which were also targets of statin drugs. This comparative design was to test the “target specificity” of the effect, which helped to distinguish whether it was a simple lipid-lowering effect or a specific effect of HMGCR inhibition.

We used the LDlink website (https://ldlink.nih.gov/?tab=ldtrait) with the European population reference panel to examine whether the selected SNPs were associated with the outcome variables and potential confounding factors. Prior to conducting the MR analysis, data harmonization was conducted to ensure consistent alignment of the effect alleles for the SNPs between both the exposure and outcome datasets.

### Cell lines and cell culture

The human PCa cell lines LNCaP and PC-3 were acquired from the Cell Bank of Shanghai Institutes for Biological Sciences (Shanghai, China). LNCaP cells were cultured in RPMI-1640 medium (Gibco, United States), while PC-3 cells in F-12 medium (Gibco, United States), both supplemented with 10% fetal bovine serum (Gibco, United States). Cells were maintained in a 37 °C incubator with 5% CO_2_, and passaged using 0.25% trypsin solution containing Ethylene Diamine Tetraacetie Acid (EDTA). All cell lines were routinely authenticated (Short Tandem Repeat (STR) profiling) and confirmed to be free of *mycoplasma* contamination.

### CCK-8 assays

Cell proliferation was assessed using a CCK-8 kit (Abcam, United States). Cells in the logarithmic growth phase were seeded into 96-well plates at a density of 1 × 10^4^ cells/mL. After 24 h of incubation to allow cell attachment, the cells were treated for an additional 24/48 h, respectively, with serial concentrations of Atorvastatin (MedChemExpress, United States) (0, 6.25, 12.5, 25, 50, 100 μM) or blank control. Subsequently, 10 μL of CCK-8 reagent was added to each well and co-cultured with the cells for 2 h in a humidified incubator at 37 °C with 5% CO_2_. The optical density (OD) at 450 nm was measured for each well, and inhibition curves of Atorvastatin were generated for each cell type. The experiment was repeated three times.

### Western blot

Protein expression levels were determined by Western blot. Total proteins were extracted from samples using RIPA lysis buffer, followed by centrifugation at 14,000 ×g for 15 min to collect the supernatant. Protein concentrations were quantified to ensure equal loading. Subsequently, proteins were separated by SDS-PAGE and transferred onto polyvinylidene difluoride (PVDF) membranes. The membranes were blocked with 5% skim milk to prevent non-specific binding and then incubated overnight at 4 °C with the following primary antibodies (all from Proteintech, China): PARP-1 (1:5,000), cleaved-PARP-1 (1:5,000), Bcl-2 (1:5,000), Bax (1:20,000), and β-actin (1:10,000). After washing, the membranes were probed with horseradish peroxidase (HRP)-conjugated secondary antibody (1:5,000; Proteintech, China) for 1 h at 37 °C. Protein signals were visualized using a chemiluminescence (ECL) detection system. The expression levels of target proteins were normalized to β-actin as an internal control. All experiments were performed in triplicate, and statistical differences between groups were assessed using t-test.

### Statistical analysis

The inverse variance weighted (IVW) method was employed, which fits a regression of SNP-outcome effects on SNP-exposure effects without an intercept, using the inverse of the outcome variance as weights. MR-Egger and Weighted Median served as supplements to the IVW.

Sensitivity analyses were conducted to evaluate the robustness of the MR results. Heterogeneity among instrumental variables was assessed using Cochran’s Q test under the inverse variance weighted (IVW) method, with a p-value <0.05 indicating significant heterogeneity and justifying the use of a random-effects model; otherwise, a common-effects model was applied. Horizontal pleiotropy was examined using MR-PRESSO and a dedicated pleiotropy test. We also performed Steiger directionality tests to rule out reverse causality. Finally, we conducted colocalization analyses to evaluate whether the genetic instruments proxying the drug targets (HMGCR and PCSK9) and PCa risk are driven by shared underlying causal genetic variants.

Statistical analyses were conducted using the “TwoSampleMR” and “coloc” package in R software version 4.2.2. Since we selected three outcome variables, we applied the Benjamini–Hochberg to adjust the P value and α < 0.05 was considered statistically significant.

## Results

### SNP selection and validation

According to our criteria, we ultimately selected 7 SNPs associated with HMGCR, 13 SNPs PCSK9 and 3 SNPs NPC1L1. All of these SNPs were strong instrumental variables (F > 10) (Supplementary Table S2).

To verify the reliability of the selected SNPs, we first examined their effects on CHD. The IVW results indicated that HMGCR inhibition decreased the risk of CHD: Per 1 standard deviation (SD, mg/dL) decrease in low-density lipoprotein cholesterol (LDL-C) was associated with a 29.3% reduction in CHD risk (GWAS ID: ieu-a-7) (odds ratio (OR) = 0.707; 95% confidence interval (CI) = 0.595–0.806; P = 2.179 × 10^−6^), and 40.9% reduction in CHD risk (GWAS ID: ebi-a-GCST000998) (OR = 0.591; 95% CI = 0.460–0.759; P = 4.015 × 10^−5^), respectively. The Weighted Median results were consistent with the IVW results: CHD (GWAS ID: ieu-a-7) (OR = 0.668; 95% CI = 0.549–0.814; P = 6.516 × 10^−5^), CHD (GWAS ID: ebi-a-GCST000998) (OR = 0.585; 95% CI = 0.436–0.784; P = 3.658 × 10^−4^).

The P-values for heterogeneity, pleiotropy, and MR-PRESSO tests were all statistically non-significant (P > 0.05), indicating the robustness of the MR (Supplementary Table S3).

The IVW results also indicated that PCSK9 inhibition decreased the risk of CHD: Per 1 standard deviation (SD, mg/dL) decrease in low-density lipoprotein cholesterol (LDL-C) was associated with a 40.3% reduction in CHD risk (GWAS ID: ieu-a-7) (odds ratio (OR) = 0.597; 95% confidence interval (CI) = 0.525–0.680; P = 7.998 × 10^−15^), and 42.6% reduction in CHD risk (GWAS ID: ebi-a-GCST000998) (OR = 0.574; 95% CI = 0.452–0.730; P = 6.104 × 10^−6^), respectively. Sensitivity analysis also indicated that the results of MR were robust (Supplementary Table S3).

Since we only selected 3 SNPs associated with NPC1L1, this limited number of IVs may increase the risk of bias in subsequent analyses. Therefore, we have excluded this target from further investigation.

### Effect of HMGCR inhibition on PCa

**FIGURE 2:**
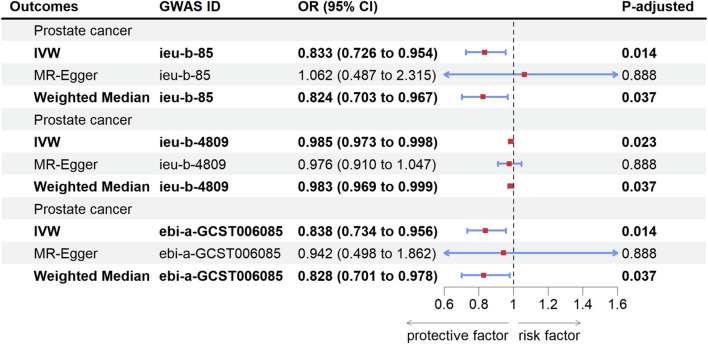
Forest plot of causal relationship between HMGCR inhibition and PCa risk. PCa, Prostate cancer; GWAS, genome-wide association study; OR, Odds ratio; CI, Confidence interval; IVW, Inverse-variance weighted method; HMGCR, 3-hydroxy-3-methylglutaryl-coenzyme A reductase.

Table 1 showed the primary results of MR. To enhance the robustness of the results, we utilized multiple GWAS datasets as results variables for PCa. The IVW results indicated that HMGCR inhibition decreased the risk of PCa. Per 1 standard deviation (SD, mg/dL) decrease in low-density lipoprotein cholesterol (LDL-C) was associated with a 16.7% reduction in PCa risk: PCa (GWAS ID: ieu-b-85) (odds ratio (OR) = 0.833; 95% confidence interval (CI) = 0.726–0.954; P-adjusted = 0.014); Per 1 SD (mg/dL) decrease in LDL-C was associated with a 1.5% reduction in PCa risk: PCa (GWAS ID: ieu-b-4809) (OR = 0.985; 95% CI = 0.973–0.998; P-adjusted = 0.023) and 16.2% reduction in PCa risk: PCa (GWAS ID: ebi-a-GCST006085) (OR = 0.838; 95% CI = 0.734–0.956; P-adjusted = 0.014). The Weighted Median results were consistent with the IVW results: PCa (GWAS ID: ieu-b-85) (OR = 0.834; 95% CI = 0.703–0.967; P-adjusted = 0.037), PCa (GWAS ID: ieu-b-4809) (OR = 0.983; 95% CI = 0.969–0.999; P-adjusted = 0.037), PCa (GWAS ID: ebi-a-GCST006085) (OR = 0.828; 95%CI = 0.734–0.956; P-adjusted = 0.037). The P-values for MR-Egger tests were all statistically non-significant (P > 0.05) (Figure 2).

**TABLE 1:** Results of Mendelian randomization to predict HMGCR inhibition on prostate cancer.

Outcomes	GWAS ID	IVW		MR-Egger		Weighted median	
		OR (95%CI)	P-adjusted	OR (95%CI)	P-adjusted	OR (95%CI)	P-adjusted
Prostate cancer	ieu-b-85	0.833 (0.726–0.954)	0.014	1.062 (0.487–2.315)	0.888	0.824 (0.703–0.967)	0.037
Prostate cancer	ieu-b-4809	0.985 (0.973–0.998)	0.023	0.976 (0.910–1.047)	0.888	0.983 (0.969–0.999)	0.037
Prostate cancer	ebi-a-GCST006085	0.838 (0.734–0.956)	0.014	0.942 (0.498–1.862)	0.888	0.828 (0.701–0.978)	0.037

HMGCR, 3-hydroxy-3-methylglutaryl-coenzyme A reductase; GWAS, Genome-Wide association study; IVW, Inverse-variance weighted method; OR, odds ratio; CI, confidence interval.

The remaining SNPs associated with exposure variables for each outcome variable after the data-harmonization steps were shown in Supplementary Table S4.

### Sensitivity analysis

The P-values for heterogeneity tests were all statistically non-significant (P > 0.05), indicating the common effect model should be used for IVW. The P-values for pleiotropy and MR-PRESSO tests were all statistically non-significant (P > 0.05), which did not support the presence of horizontal pleiotropy (Table 2).

**TABLE 2:** Mendelian randomization in sensitivity analysis to predict HMGCR inhibition on prostate cancer.

Outcomes	GWAS ID	Pleiotropy		Heterogeneity		MR-PRESSO
		Intercept	P-value	Q	P-value	P-value
Prostate cancer	ieu-b-85	0.015	0.568	3.945	0.557	0.622
Prostate cancer	ieu-b-4809	0.000	0.796	3.570	0.613	0.693
Prostate cancer	ebi-a-GCST006085	0.007	0.746	4.066	0.668	0.715

HMGCR, 3-hydroxy-3-methylglutaryl-coenzyme A reductase; GWAS, Genome-Wide association study.

The results of the leave-one-out sensitivity analysis, forest plots, scatter plots, and funnel plots for the association between statins use and PC were presented in Supplementary Figures S1–S4, respectively, demonstrating the appropriateness of the selected SNPs and the robustness of the MR findings.

### Effect of PCSK9 inhibition on PCa

To assess whether the effects of statins on prostate cancer are due to HMGCR inhibition specifically or merely a consequence of lipid-lowering, we also performed Mendelian randomization analyses using genetic variants associated with other statin drug targets (PCSK9).

The IVW results indicated that PCSK9 inhibition decreased the risk of PCa. Per 1 standard deviation (SD, mg/dL) decrease in low-density lipoprotein cholesterol (LDL-C) was associated with a 19.2% reduction in PCa risk (GWAS ID: ieu-b-85) (odds ratio (OR) = 0.808; 95% confidence interval (CI) = 0.754–0.891; P-adjusted = 1.737 × 10^−05^) and 18.8% reduction in PCa risk (GWAS ID: ebi-a-GCST006085) (OR = 0.812; 95% CI = 0.739–0.894; P-adjusted = 1.737 × 10^−05^). When GWAS datasets for PCa (GWAS ID: ieu-b-4809) used as outcome variables exhibit horizontal pleiotropy, the analytical results based on these datasets were excluded.

Sensitivity analysis also indicated that the results of MR were robust (Supplementary Table S5).

### Steiger directionality tests

We also performed Steiger directionality tests to rule out the potential effect of PCa on LDL. The results of the overall Steiger directionality test, indicated the absence of reverse causality (P < 0.05) (Supplementary Table S6). Furthermore, the SNP-specific Steiger filtering test results demonstrated that all selected SNPs were appropriate and likewise confirm the lack of reverse causality (P < 0.05) (Supplementary Table S4).

### Results of colocalization analyses

We conducted colocalization analyses to evaluate whether the genetic instruments proxying the drug targets (HMGCR and PCSK9) and PCa risk are driven by shared underlying causal genetic variants. However, the results indicated that no shared genetic variants were found in either the HMGCR region or the PCSK9 region (Supplementary Table S7).

### Results of cell experiments

**FIGURE 3:**
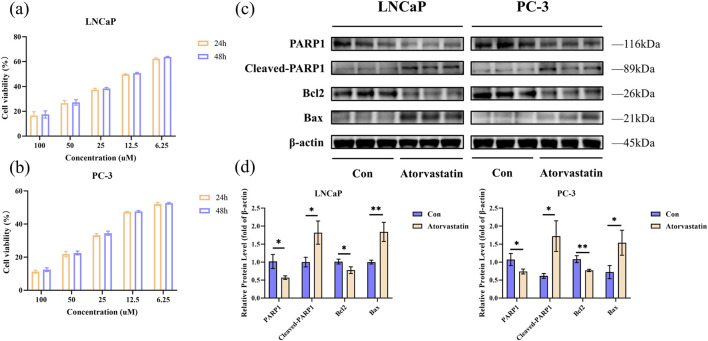
Results of cell experiments. The viability was measured by CCK-8 assay for LNCaP **(a)** and PC-3 **(b)**; Analysis of apoptosis by Western blot in LNCaP and PC-3 cells treated with Atorvastatin for 24 h **(c)**; Quantitative analysis of Western blot **(d)**. The values were showed as the mean ± SEM (n = 3). *p < 0.05, **p < 0.01. CCK-8, Cell counting kit-8.

To determine the optimal experimental concentrations of Atorvastatin for subsequent experiments, the effects of 24/48-h treatment with varying concentrations of Atorvastatin were evaluated using the CCK-8 proliferation assay in LNCaP and PC-3 cells. Treatment with increasing doses of Atorvastatin for 24/48 h, respectively, inhibited the proliferation of PCa cells in a dose-dependent manner (Figures 3a,b). According to the inhibition curve, the half maximal inhibitory concentration (IC50) of Atorvastatin for LNCaP and PC-3 was 13.7 μM and 8.5 μM, respectively. Therefore, we selected 10 μM Atorvastatin for the subsequent studies.

The apoptotic response of LNCaP and PC-3 cells following Atorvastatin treatment was assessed using Western blot. We detected the expression levels of four apoptosis-related proteins. The results indicated that a 24-h intervention with Atorvastatin promoted the cleavage of PARP1 protein, suppressed Bcl2 expression, and enhanced Bax expression (Figure 3c). Figure 3d showed the quantification of the Western blot results (P < 0.05).

## Discussion

The effect of statins on PCa is an important clinical issue, but the results of various studies have been inconsistent. Previous studies have often been limited by confounding factors and reverse causality, potentially compromising the accuracy of their findings. In contrast, MR effectively mitigates these limitations by minimizing confounding and avoiding reverse causality, making it a reliable method for studying causal relationships.

Previous retrospective studies have explored the therapeutic effect of statin use on prostate cancer: Meta-analysis results by Tan et al. showed no type of statins affected the risk of PCa (Tan et al., 2017); Research by Alfaqih et al. demonstrated that statins use did not prevent PCa (Alfaqih et al., 2017); Research by Kumar et al. showed that patients using statins underwent more PCa screenings, and the increased rate of early screening improved PCa mortality; however, when screening utilization was taken into account, statins did not improve PCa mortality (Kumar et al., 2020).

Our research results of MR indicated that statins use could reduce the risk of PCa, which was consistent with the following findings: Case-control study by Shannon et al. demonstrated that statins could reduce the risk of PCa, particularly more aggressive PCa (Shannon et al., 2005; Craig et al., 2022); Research by Van et al. showed that statins can reduce the risk of metastatic PCa and PCa mortality (Van Rompay et al., 2019); Research by Yang et al. indicated that combining statins with castration therapy could reduce all-cause mortality and cancer-specific mortality in PCa patients (Yang et al., 2020; Tiwari and Fleshner, 2022). We selected two PCa cell lines (LNCaP and PC-3) for *in vitro* experiments to further enhance the robustness of our MR results. Both of these cell lines are human PCa cells and are widely used in *in vitro* experiments. Using two distinct cell lines for the in vitrostudies adds persuasiveness to the findings. Our cell experiments also demonstrated that Atorvastatin intervention can promote apoptosis and reduce invasion in LNCaP and PC-3 cells. While *in vitro* experiments cannot fully replicate the complex tumor microenvironment and heterogeneity found in the human body, they provide evidence that, to some extent, supports the findings from the MR analysis.

Statins may exert anti-cancer effects through the following mechanisms: Statins may reduce the risk of PCa by inhibiting cholesterol synthesis, thereby suppressing androgen production and consequently inhibiting the progression of PCa (Mostaghel, 2021). Additionally, statins could trigger tumor cell-specific apoptosis (Wong et al., 2001; Longo et al., 2019). Research by Sekine et al. suggested that statin use downregulated DeoxyriboNucleic Acid (DNA) repair genes in PCa cells, and when combined with polymerase inhibitors, it could suppress the proliferation of castration-resistant PCa cells (Sekine et al., 2025). We identified HMGCR-related SNPs and confirmed their protective effect against CHD, indicating that these SNPs serve as genetic instruments representing lipid-lowering effects. Subsequently, Mendelian randomization analysis revealed that HMGCR inhibition and PCSK9 inhibition were associated with a reduced risk of PCa, suggesting that HMGCR inhibition may exert its protective effect against PCa through lipid-lowering mechanisms.

When evaluating the effect of PCSK9 inhibition on PCa, GWAS datasets for PCa (GWAS ID: ieu-b-4809) used as outcome variables exhibit horizontal pleiotropy. We excluded the outlier SNPs and repeated the MR analyses, but horizontal pleiotropy was still detected. The persistence of pleiotropy may be attributed to additional genetic associations or linkage disequilibrium between certain SNPs and the PCa GWAS datasets. Given this irreducible horizontal pleiotropy, the analytical results based on the datasets were excluded.

After confirming the causal effects of HMGCR inhibition and PCSK9 inhibition on PCa, we further employed colocalization analysis to investigate whether this association was driven by shared genetic variants. However, our colocalization results indicated that no shared genetic variants were found in either the HMGCR region or the PCSK9 region. Colocalization analysis typically tests a single causal variant model. If the association at a particular locus is driven by multiple closely linked but distinct causal variants, colocalization methods may fail to detect such a complex genetic structure, potentially leading to negative results. This interesting result may indicate that the relationship is more complex than a single mediating pathway, possibly involving pleiotropic mechanisms independent of LDL-lowering, such as statins may exert anti-tumor effects through anti-inflammatory mechanisms or by modulating the gut microbiota to inhibit cancer proliferation (Jiang et al., 2021; Han et al., 2023). Future research employing tissue-specific functional genomics and experimental models is warranted to disentangle the precise mechanisms linking HMGCR inhibition to PCa risk reduction. Our findings suggested that HMGCR inhibition may exert effects beyond lipid-lowering, pointing to new directions for future mechanistic research.

Some limitations of this study should be noted: Firstly, we were unable to categorize the types, dosages, and duration of statin use; Secondly, we could not perform subgroup analyses based on different PCa outcomes, stages and pathological types. Meanwhile, the genetic variants proxy lifelong HMGCR inhibition, which may not perfectly mimic the effect of mid-to-late life pharmacological statin use in terms of effect size, duration, or biological pathway; Lastly, the mechanisms by which statins use alleviated risk of PCa required further investigation.

## Conclusion

HMGCR inhibition reduces the risk of PCa, and this protective effect is independent of its lipid-lowering action. Our findings provide strong genetic support for initiating RCTs to investigate the therapeutic potential of statins for partial PCa patients.

## Data Availability

The original contributions presented in the study are included in the article/Supplementary Material, further inquiries can be directed to the corresponding author.
