# Supplementary material for: Association of genetically instrumented HMGCR inhibition with the therapeutic role of prostate cancer: a Mendelian randomization study and supporting in vitro experiments
Source: Front Pharmacol. 2026 Jan 12;16:1701869. doi: 10.3389/fphar.2025.1701869 (PMC12833389; doi:10.3389/fphar.2025.1701869)
Supplement: Supplementary file 1 [file DataSheet1.docx]

| Supplementary-Table 1. Baseline characteristics of low-density lipoprotein, coronary heart disease and prostate cancer | | | | | | | | | |
| --- | --- | --- | --- | --- | --- | --- | --- | --- | --- |
| Trait | GWAS ID | Consortium | Year | Population | Unit | Number of case | Number of control | Sample Size | Number of SNPs |
| Low-density lipoprotein | ieu-a-300 | GLGC | 2013 | Mixed | SD (mg/dL) | —— | —— | 173,082 | 2,437,752 |
| Coronary heart disease | ieu-a-7 | CARDIoGRAM | 2015 | Mixed | logOR | 60,801 | 123,504 | 184,305 | 9,455,779 |
| Coronary heart disease | ebi-a-GCST000998 | —— | 2011 | European | logOR | 22,233 | 64,762 | 86,995 | 2,415,020 |
| Prostate cancer | ieu-b-85 | PRACTICAL | 2018 | European | —— | 79,148 | 61,106 | 140,254 | 20,346,368 |
| Prostate cancer | ieu-b-4809 | UK Biobank | 2021 | European | —— | 9,132 | 173,493 | 182,625 | —— |
| Prostate cancer | ebi-a-GCST006085 | —— | 2018 | European | —— | 79,148 | 61,106 | 140,254 | 19,733,911 |
| GWAS, Genome-Wide association study; SNPs, Single nucleotide polymorphisms. | | | | | | | | | |

| Supplementary-Table 2. Selection of Single nucleotide polymorphisms | | | | | | | | | |
| --- | --- | --- | --- | --- | --- | --- | --- | --- | --- |
| Drug targeting | SNPs | Chr | EA | NEA | EAF | BETA | SE | P-value | F |
| HMGCR | rs3857388 | 5 | C | T | 0.1280 | 0.0421 | 0.0059 | 2.20090E-11 | 51 |
| HMGCR | rs10515198 | 5 | A | G | 0.1029 | 0.0599 | 0.0061 | 5.98963E-22 | 96 |
| HMGCR | rs12659791 | 5 | C | T | 0.1557 | 0.0433 | 0.0050 | 1.42200E-18 | 75 |
| HMGCR | rs72633962 | 5 | C | T | 0.1412 | 0.0600 | 0.0072 | 3.32736E-15 | 69 |
| HMGCR | rs3804231 | 5 | A | G | 0.1319 | 0.0642 | 0.0053 | 1.88408E-29 | 147 |
| HMGCR | rs10066707 | 5 | A | G | 0.4169 | 0.0497 | 0.0054 | 2.97303E-19 | 85 |
| HMGCR | rs12916 | 5 | C | T | 0.4314 | 0.0733 | 0.0038 | 7.79112E-78 | 372 |
| PCSK9 | rs2479394 | 1 | G | A | 0.2850 | 0.0390 | 0.0040 | 1.58000E-19 | 95 |
| PCSK9 | rs11206510 | 1 | T | C | 0.8460 | 0.0830 | 0.0050 | 2.38000E-53 | 276 |
| PCSK9 | rs2495495 | 1 | T | C | 0.1350 | 0.0340 | 0.0060 | 3.52000E-08 | 32 |
| PCSK9 | rs2479409 | 1 | G | A | 0.3330 | 0.0640 | 0.0040 | 2.52000E-50 | 256 |
| PCSK9 | rs11591147 | 1 | G | T | 0.9830 | 0.4970 | 0.0180 | 8.58000E-143 | 762 |
| PCSK9 | rs4927193 | 1 | T | C | 0.8690 | 0.0350 | 0.0060 | 4.27000E-11 | 34 |
| PCSK9 | rs11206514 | 1 | A | C | 0.6110 | 0.0510 | 0.0040 | 9.95000E-33 | 163 |
| PCSK9 | rs572512 | 1 | T | C | 0.3460 | 0.0480 | 0.0050 | 5.31000E-26 | 92 |
| PCSK9 | rs585131 | 1 | T | C | 0.8150 | 0.0640 | 0.0050 | 2.70000E-35 | 164 |
| PCSK9 | rs12067569 | 1 | A | G | 0.0340 | 0.0890 | 0.0100 | 1.97000E-17 | 79 |
| PCSK9 | rs10493176 | 1 | T | G | 0.8850 | 0.0780 | 0.0100 | 2.54000E-14 | 61 |
| PCSK9 | rs11583974 | 1 | A | G | 0.0300 | 0.0650 | 0.0120 | 3.95000E-09 | 29 |
| PCSK9 | rs2495477 | 1 | T | C | NA | 0.0640 | 0.0050 | 7.29000E-30 | 164 |
| NPC1L1 | rs2073547 | 7 | G | A | 0.1939 | 0.0485 | 0.0049 | 1.92309E-21 | 98 |
| NPC1L1 | rs217386 | 7 | A | G | 0.4077 | -0.0363 | 0.0038 | 1.20393E-19 | 91 |
| NPC1L1 | rs7791240 | 7 | C | T | 0.0910 | 0.0425 | 0.0065 | 1.83802E-10 | 43 |
| HMGCR, 3-hydroxy-3-methylglutaryl-coenzyme A reductase; PCSK9, Proprotein convertase subtilisin/kexin type 9; NPC1L1, Niemann–Pick C1-Like 1; SNPs, Single nucleotide polymorphisms; Chr, Chromosome; EA, Effect allele; NEA, Non effect allele; EAF, Effect allele frequency; SE, Standard error. | | | | | | | | | |
|  |  |  |  |  |  |  |  |  |  |

| Supplementary-Table 3. Mendelian randomization analysis to predict HMGCR and PCSK9 inhibition on CHD outcomes | | | | | | | | | | | | | |
| --- | --- | --- | --- | --- | --- | --- | --- | --- | --- | --- | --- | --- | --- |
| Outcome | Drug targeting | GWAS ID | IVW | | MR-Egger | | Weighted Median | | Pleiotropy | | Heterogeneity | | MR-PRESSO |
|  |  |  | OR(95%CI) | P-value | OR(95%CI) | P-value | OR(95%CI) | P-value | Intercept | P-value | Q | P-value | P-value |
| Coronary heart disease | HMGCR | ieu-a-7 | 0.707(0.595-0.806) | 2.179E-06 | 0.426(0.194-0.935) | 8.689E-02 | 0.668(0.549-0.814) | 6.516E-05 | -0.029 | 0.272 | 4.685 | 0.585 | 0.584 |
| Coronary heart disease | HMGCR | ebi-a-GCST000998 | 0.591(0.460-0.759) | 4.015E-05 | 0.424(0.122-1.468) | 2.471E-01 | 0.585(0.436-0.786) | 3.658E-04 | -0.020 | 0.621 | 2.655 | 0.793 | 0.821 |
| Coronary heart disease | PCSK9 | ieu-a-7 | 0.597(0.525-0.680) | 7.998E-15 | 0.575(0.436-0.759) | 2.468E-03 | 0.593(0.504-0.697) | 2.318E-10 | -0.003 | 0.768 | 17.130 | 0.145 | 0.187 |
| Coronary heart disease | PCSK9 | ebi-a-GCST000998 | 0.574(0.452-0.730) | 6.104E-06 | 0.374(0.162-0.864) | 5.498E-02 | 0.561(0.407-0.773) | 4.214E-04 | -0.027 | 0.330 | 6.084 | 0.638 | 0.637 |
| HMGCR, 3-hydroxy-3-methylglutaryl-coenzyme A reductase; PCSK9, Proprotein convertase subtilisin/kexin type 9; CHD, Coronary heart disease; GWAS, Genome-Wide association study; IVW, Inverse-variance weighted method; OR, Odds ratio; CI, Confidence interval. | | | | | | | | | | | | | |

| Supplementary-Table 4. Single nucleotide polymorphisms used as instrumental variables in the Mendelian randomization analysis | | | | | | | | | | | | | |
| --- | --- | --- | --- | --- | --- | --- | --- | --- | --- | --- | --- | --- | --- |
| Drug targeting | GWAS ID of Outcomes | SNPs | EA | NEA | EAF | BETA | SE | P-value | F | rsq.exposure | rsq.outcome | steiger_dir | steiger_pval |
| HMGCR | ieu-b-85 | rs3857388 | C | T | 0.1280 | 0.0421 | 0.0059 | 2.20090E-11 | 51 | 2.941E-04 | 7.018E-06 | TRUE | 5.428E-05 |
| HMGCR | ieu-b-85 | rs10515198 | A | G | 0.1029 | 0.0599 | 0.0061 | 5.98963E-22 | 96 | 5.568E-04 | 5.602E-06 | TRUE | 3.414E-09 |
| HMGCR | ieu-b-85 | rs72633962 | C | T | 0.1412 | 0.0600 | 0.0072 | 3.32736E-15 | 69 | 4.011E-04 | 6.295E-06 | TRUE | 1.079E-06 |
| HMGCR | ieu-b-85 | rs3804231 | A | G | 0.1319 | 0.0642 | 0.0053 | 1.88408E-29 | 147 | 8.470E-04 | 9.919E-06 | TRUE | 4.959E-13 |
| HMGCR | ieu-b-85 | rs10066707 | A | G | 0.4169 | 0.0497 | 0.0054 | 2.97303E-19 | 85 | 4.892E-04 | 2.852E-05 | TRUE | 3.002E-06 |
| HMGCR | ieu-b-85 | rs12916 | C | T | 0.4314 | 0.0733 | 0.0038 | 7.79112E-78 | 372 | 2.145E-03 | 2.010E-05 | TRUE | 2.218E-31 |
| HMGCR | ieu-b-4809 | rs3857388 | C | T | 0.1280 | 0.0421 | 0.0059 | 2.20090E-11 | 51 | 2.941E-04 | 4.355E-07 | TRUE | 8.838E-07 |
| HMGCR | ieu-b-4809 | rs10515198 | A | G | 0.1029 | 0.0599 | 0.0061 | 5.98963E-22 | 96 | 5.568E-04 | 5.168E-06 | TRUE | 2.048E-10 |
| HMGCR | ieu-b-4809 | rs72633962 | C | T | 0.1412 | 0.0600 | 0.0072 | 3.32736E-15 | 69 | 4.011E-04 | 1.034E-05 | TRUE | 5.378E-07 |
| HMGCR | ieu-b-4809 | rs3804231 | A | G | 0.1319 | 0.0642 | 0.0053 | 1.88408E-29 | 147 | 8.470E-04 | 1.103E-05 | TRUE | 1.491E-14 |
| HMGCR | ieu-b-4809 | rs10066707 | A | G | 0.4169 | 0.0497 | 0.0054 | 2.97303E-19 | 85 | 4.892E-04 | 6.641E-06 | TRUE | 5.679E-09 |
| HMGCR | ieu-b-4809 | rs12916 | C | T | 0.4314 | 0.0733 | 0.0038 | 7.79112E-78 | 372 | 2.145E-03 | 1.439E-05 | TRUE | 7.105E-37 |
| HMGCR | ebi-a-GCST006085 | rs3857388 | C | T | 0.1280 | 0.0421 | 0.0059 | 2.20090E-11 | 51 | 2.941E-04 | 7.018E-06 | TRUE | 5.428E-05 |
| HMGCR | ebi-a-GCST006085 | rs10515198 | A | G | 0.1029 | 0.0599 | 0.0061 | 5.98963E-22 | 96 | 5.568E-04 | 5.602E-06 | TRUE | 3.414E-09 |
| HMGCR | ebi-a-GCST006085 | rs12659791 | C | T | 0.1557 | 0.0433 | 0.0050 | 1.42200E-18 | 75 | 4.331E-04 | 9.602E-07 | TRUE | 3.376E-08 |
| HMGCR | ebi-a-GCST006085 | rs72633962 | C | T | 0.1412 | 0.0600 | 0.0072 | 3.32736E-15 | 69 | 4.011E-04 | 6.295E-06 | TRUE | 1.079E-06 |
| HMGCR | ebi-a-GCST006085 | rs3804231 | A | G | 0.1319 | 0.0642 | 0.0053 | 1.88408E-29 | 147 | 8.470E-04 | 9.919E-06 | TRUE | 4.959E-13 |
| HMGCR | ebi-a-GCST006085 | rs10066707 | A | G | 0.4169 | 0.0497 | 0.0054 | 2.97303E-19 | 85 | 4.892E-04 | 2.852E-05 | TRUE | 3.002E-06 |
| HMGCR | ebi-a-GCST006085 | rs12916 | C | T | 0.4314 | 0.0733 | 0.0038 | 7.79112E-78 | 372 | 2.145E-03 | 2.010E-05 | TRUE | 2.218E-31 |
| PCSK9 | ieu-b-85 | rs2479394 | G | A | 0.2850 | 0.0390 | 0.0040 | 1.58000E-19 | 95 | 5.489E-04 | 4.039E-06 | TRUE | 2.475E-09 |
| PCSK9 | ieu-b-85 | rs11206510 | T | C | 0.8460 | 0.0830 | 0.0050 | 2.38000E-53 | 276 | 1.590E-03 | 3.163E-05 | TRUE | 1.460E-21 |
| PCSK9 | ieu-b-85 | rs2479409 | G | A | 0.3330 | 0.0640 | 0.0040 | 2.52000E-50 | 256 | 1.477E-03 | 1.768E-05 | TRUE | 1.549E-21 |
| PCSK9 | ieu-b-85 | rs11591147 | G | T | 0.9830 | 0.4970 | 0.0180 | 8.58000E-143 | 762 | 4.385E-03 | 2.164E-05 | TRUE | 4.899E-66 |
| PCSK9 | ieu-b-85 | rs4927193 | T | C | 0.8690 | 0.0350 | 0.0060 | 4.27000E-11 | 34 | 1.966E-04 | 2.432E-06 | TRUE | 5.233E-04 |
| PCSK9 | ieu-b-85 | rs11206514 | A | C | 0.6110 | 0.0510 | 0.0040 | 9.95000E-33 | 163 | 9.384E-04 | 7.458E-06 | TRUE | 7.924E-15 |
| PCSK9 | ieu-b-85 | rs572512 | T | C | 0.3460 | 0.0480 | 0.0050 | 5.31000E-26 | 92 | 5.322E-04 | 5.070E-05 | TRUE | 8.984E-06 |
| PCSK9 | ieu-b-85 | rs585131 | T | C | 0.8150 | 0.0640 | 0.0050 | 2.70000E-35 | 164 | 9.457E-04 | 4.440E-05 | TRUE | 1.979E-11 |
| PCSK9 | ieu-b-85 | rs12067569 | A | G | 0.0340 | 0.0890 | 0.0100 | 1.97000E-17 | 79 | 4.574E-04 | 3.780E-06 | TRUE | 6.202E-08 |
| PCSK9 | ieu-b-85 | rs10493176 | T | G | 0.8850 | 0.0780 | 0.0100 | 2.54000E-14 | 61 | 3.514E-04 | 5.143E-06 | TRUE | 4.497E-06 |
| PCSK9 | ieu-b-85 | rs11583974 | A | G | 0.0300 | 0.0650 | 0.0120 | 3.95000E-09 | 29 | 1.695E-04 | 5.422E-05 | TRUE | 1.154E-01 |
| PCSK9 | ieu-b-85 | rs2495477 | T | C | NA | 0.0640 | 0.0050 | 7.29000E-30 | 164 | 9.457E-04 | 4.697E-05 | TRUE | 2.838E-11 |
| PCSK9 | ieu-b-4809 | rs2479394 | G | A | 0.2850 | 0.0390 | 0.0040 | 1.58000E-19 | 95 | 5.489E-04 | 2.425E-05 | TRUE | 3.436E-08 |
| PCSK9 | ieu-b-4809 | rs11206510 | T | C | 0.8460 | 0.0830 | 0.0050 | 2.38000E-53 | 276 | 1.590E-03 | 9.642E-07 | TRUE | 4.194E-31 |
| PCSK9 | ieu-b-4809 | rs2479409 | G | A | 0.3330 | 0.0640 | 0.0040 | 2.52000E-50 | 256 | 1.477E-03 | 3.592E-06 | TRUE | 1.196E-27 |
| PCSK9 | ieu-b-4809 | rs11591147 | G | T | 0.9830 | 0.4970 | 0.0180 | 8.58000E-143 | 762 | 4.385E-03 | 4.746E-06 | TRUE | 1.715E-81 |
| PCSK9 | ieu-b-4809 | rs4927193 | T | C | 0.8690 | 0.0350 | 0.0060 | 4.27000E-11 | 34 | 1.966E-04 | 1.129E-05 | TRUE | 1.483E-03 |
| PCSK9 | ieu-b-4809 | rs11206514 | A | C | 0.6110 | 0.0510 | 0.0040 | 9.95000E-33 | 163 | 9.384E-04 | 4.480E-07 | TRUE | 4.080E-19 |
| PCSK9 | ieu-b-4809 | rs572512 | T | C | 0.3460 | 0.0480 | 0.0050 | 5.31000E-26 | 92 | 5.322E-04 | 5.245E-06 | TRUE | 5.815E-10 |
| PCSK9 | ieu-b-4809 | rs585131 | T | C | 0.8150 | 0.0640 | 0.0050 | 2.70000E-35 | 164 | 9.457E-04 | 1.485E-06 | TRUE | 1.288E-18 |
| PCSK9 | ieu-b-4809 | rs12067569 | A | G | 0.0340 | 0.0890 | 0.0100 | 1.97000E-17 | 79 | 4.574E-04 | 2.545E-05 | TRUE | 1.101E-06 |
| PCSK9 | ieu-b-4809 | rs10493176 | T | G | 0.8850 | 0.0780 | 0.0100 | 2.54000E-14 | 61 | 3.514E-04 | 5.824E-05 | TRUE | 9.213E-04 |
| PCSK9 | ieu-b-4809 | rs11583974 | A | G | 0.0300 | 0.0650 | 0.0120 | 3.95000E-09 | 29 | 1.695E-04 | 1.096E-06 | TRUE | 3.584E-04 |
| PCSK9 | ieu-b-4809 | rs2495477 | T | C | NA | 0.0640 | 0.0050 | 7.29000E-30 | 164 | 9.457E-04 | 2.251E-08 | TRUE | 7.148E-20 |
| PCSK9 | ebi-a-GCST006085 | rs2479394 | G | A | 0.2850 | 0.0390 | 0.0040 | 1.58000E-19 | 95 | 5.489E-04 | 4.039E-06 | TRUE | 2.475E-09 |
| PCSK9 | ebi-a-GCST006085 | rs11206510 | T | C | 0.8460 | 0.0830 | 0.0050 | 2.38000E-53 | 276 | 1.590E-03 | 3.163E-05 | TRUE | 1.460E-21 |
| PCSK9 | ebi-a-GCST006085 | rs2495495 | T | C | 0.1350 | 0.0340 | 0.0060 | 3.52000E-08 | 32 | 1.855E-04 | 1.003E-06 | TRUE | 4.441E-04 |
| PCSK9 | ebi-a-GCST006085 | rs2479409 | G | A | 0.3330 | 0.0640 | 0.0040 | 2.52000E-50 | 256 | 1.477E-03 | 1.768E-05 | TRUE | 1.549E-21 |
| PCSK9 | ebi-a-GCST006085 | rs11591147 | G | T | 0.9830 | 0.4970 | 0.0180 | 8.58000E-143 | 762 | 4.385E-03 | 2.164E-05 | TRUE | 4.899E-66 |
| PCSK9 | ebi-a-GCST006085 | rs4927193 | T | C | 0.8690 | 0.0350 | 0.0060 | 4.27000E-11 | 34 | 1.966E-04 | 2.432E-06 | TRUE | 5.233E-04 |
| PCSK9 | ebi-a-GCST006085 | rs11206514 | A | C | 0.6110 | 0.0510 | 0.0040 | 9.95000E-33 | 163 | 9.384E-04 | 7.458E-06 | TRUE | 7.924E-15 |
| PCSK9 | ebi-a-GCST006085 | rs572512 | T | C | 0.3460 | 0.0480 | 0.0050 | 5.31000E-26 | 92 | 5.322E-04 | 5.070E-05 | TRUE | 8.984E-06 |
| PCSK9 | ebi-a-GCST006085 | rs585131 | T | C | 0.8150 | 0.0640 | 0.0050 | 2.70000E-35 | 164 | 9.457E-04 | 4.440E-05 | TRUE | 1.979E-11 |
| PCSK9 | ebi-a-GCST006085 | rs12067569 | A | G | 0.0340 | 0.0890 | 0.0100 | 1.97000E-17 | 79 | 4.574E-04 | 3.780E-06 | TRUE | 6.202E-08 |
| PCSK9 | ebi-a-GCST006085 | rs10493176 | T | G | 0.8850 | 0.0780 | 0.0100 | 2.54000E-14 | 61 | 3.514E-04 | 5.143E-06 | TRUE | 4.497E-06 |
| PCSK9 | ebi-a-GCST006085 | rs11583974 | A | G | 0.0300 | 0.0650 | 0.0120 | 3.95000E-09 | 29 | 1.695E-04 | 5.422E-05 | TRUE | 1.154E-01 |
| PCSK9 | ebi-a-GCST006085 | rs2495477 | T | C | NA | 0.0640 | 0.0050 | 7.29000E-30 | 164 | 9.457E-04 | 4.697E-05 | TRUE | 2.838E-11 |
| GWAS, Genome-Wide association study; SNPs, Single nucleotide polymorphisms; EA, Effect allele; NEA, Non effect allele; SE, Standard error; HMGCR, 3-hydroxy-3-methylglutaryl-coenzyme A reductase; PCSK9, Proprotein convertase subtilisin/kexin type 9. | | | | | | | | | | | | | |
|  |  |  |  |  |  |  |  |  |  |  |  |  |  |

| Supplementary-Table 5. Results of Mendelian randomization to predict PCSK9 inhibition on prostate cancer | | | | | | | | | | | | |
| --- | --- | --- | --- | --- | --- | --- | --- | --- | --- | --- | --- | --- |
| Outcomes | GWAS ID | IVW |  | MR-Egger |  | Weighted Median |  | Pleiotropy | | Heterogeneity | | MR-PRESSO |
|  |  | OR(95%CI) | P-adjusted | OR(95%CI) | P-adjusted | OR(95%CI) | P-adjusted | Intercept | P-value | Q | P-value | P-value |
| Prostate cancer | ieu-b-85 | 0.808(0.754-0.891) | 1.737E-05 | 0.906(0.784-1.047) | 2.092E-01 | 0.847(0.759-0.945) | 4.766E-03 | 0.011 | 0.084 | 15.145 | 0.176 | 0.213 |
| Prostate cancer | ebi-a-GCST006085 | 0.812(0.739-0.894) | 1.737E-05 | 0.890(0.767-1.034) | 2.092E-01 | 0.850(0.758-0.951) | 4.766E-03 | 0.009 | 0.159 | 16.085 | 0.187 | 0.209 |
| PCSK9, Proprotein convertase subtilisin/kexin type 9; GWAS, Genome-Wide association study; IVW, Inverse-variance weighted method; OR, Odds ratio; CI, Confidence interval. | | | | | | | | | | | | |

| Supplementary-Table 6. Steiger test to evaluate the effect of genetically predicted HMGCR and PCSK9 inhibition on prostate cancer | | | | | |
| --- | --- | --- | --- | --- | --- |
| Drug targeting | GWAS ID of Outcomes | snp_r2.exposure | snp_r2.outcome | correct_causal_direction | steiger_pval |
| HMGCR | ieu-b-85 | 4.364E-03 | 7.731E-05 | TRUE | 2.177E-57 |
| HMGCR | ieu-b-4809 | 4.364E-03 | 4.822E-05 | TRUE | 9.957E-70 |
| HMGCR | ebi-a-GCST006085 | 4.811E-03 | 7.826E-05 | TRUE | 6.922E-64 |
| PCSK9 | ieu-b-85 | 1.115E-02 | 2.893E-04 | TRUE | 1.898E-135 |
| PCSK9 | ieu-b-4809 | 1.115E-02 | 1.371E-04 | TRUE | 7.949E-174 |
| PCSK9 | ebi-a-GCST006085 | 1.133E-02 | 2.903E-04 | TRUE | 6.971E-138 |
| GWAS, Genome-Wide association study; HMGCR, 3-hydroxy-3-methylglutaryl-coenzyme A reductase; PCSK9, Proprotein convertase subtilisin/kexin type 9. | | | | | |
|  |  |  |  |  |  |

| Supplementary-Table 7. Results of Colocalization analyses | | | | | | |
| --- | --- | --- | --- | --- | --- | --- |
| Drug targeting | GWAS ID of outcomes | PPH0 | PPH1 | PPH2 | PPH3 | PPH4 |
| HMGCR | ieu-b-85 | 6.03E-79 | 9.60E-01 | 1.09E-80 | 1.73E-02 | 2.29E-02 |
| HMGCR | ieu-b-4809 | 6.26E-79 | 9.97E-01 | 1.52E-81 | 2.42E-03 | 7.23E-04 |
| HMGCR | ebi-a-GCST006085 | 6.04E-79 | 9.59E-01 | 1.16E-80 | 1.85E-02 | 2.29E-02 |
| PCSK9 | ieu-b-85 | 3.72E-160 | 9.15E-01 | 8.65E-162 | 2.12E-02 | 6.38E-02 |
| PCSK9 | ieu-b-4809 | 4.05E-160 | 9.97E-01 | 5.32E-163 | 1.31E-03 | 2.12E-03 |
| PCSK9 | ebi-a-GCST006085 | 3.72E-160 | 9.14E-01 | 9.23E-162 | 2.26E-02 | 6.38E-02 |
| GWAS, Genome-Wide association study; PPH, Posterior Probability for Hypothesis; HMGCR, 3-hydroxy-3-methylglutaryl-coenzyme A reductase; PCSK9, Proprotein convertase subtilisin/kexin type 9. | | | | | | |
|  |  |  |  |  |  |  |


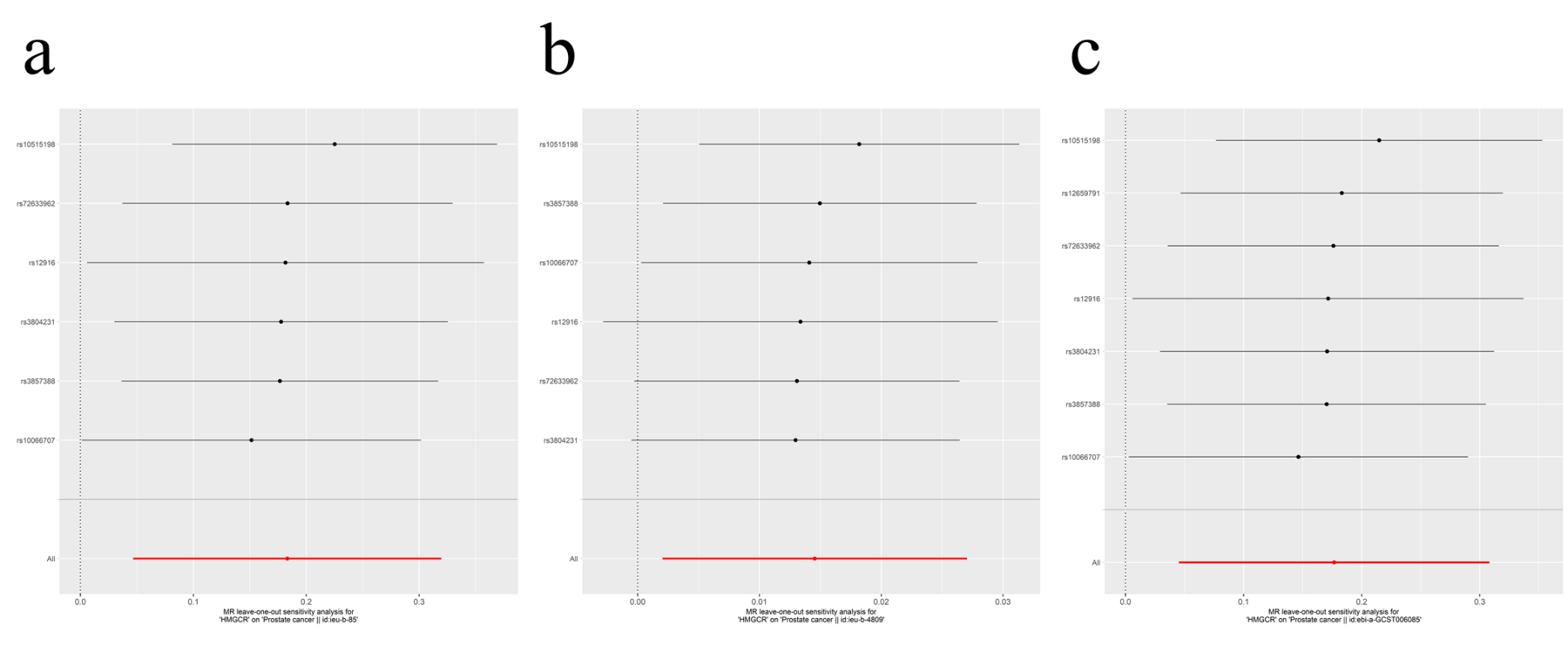


Supplementary-Figure 1. Results of Leave‐one‐out method: (a) Prostate cancer (GWAS ID: ieu-b-85); (b) Prostate cancer (GWAS ID: ieu-b-4809); (c) Prostate cancer (GWAS ID: ebi-a-GCST006085). GWAS, Genome-Wide association study.


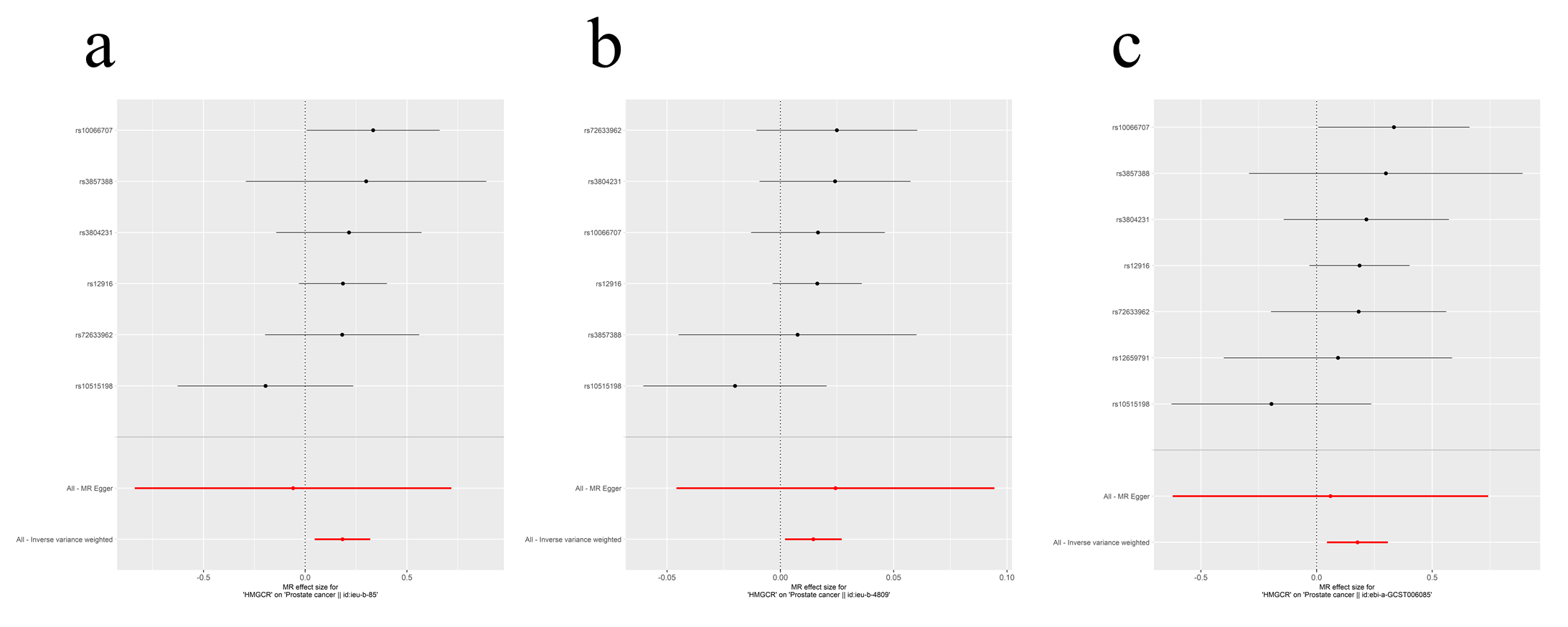


Supplementary-Figure 2. Results of forest plot: (a) Prostate cancer (GWAS ID: ieu-b-85); (b) Prostate cancer (GWAS ID: ieu-b-4809); (c) Prostate cancer (GWAS ID: ebi-a-GCST006085). GWAS, Genome-Wide association study.


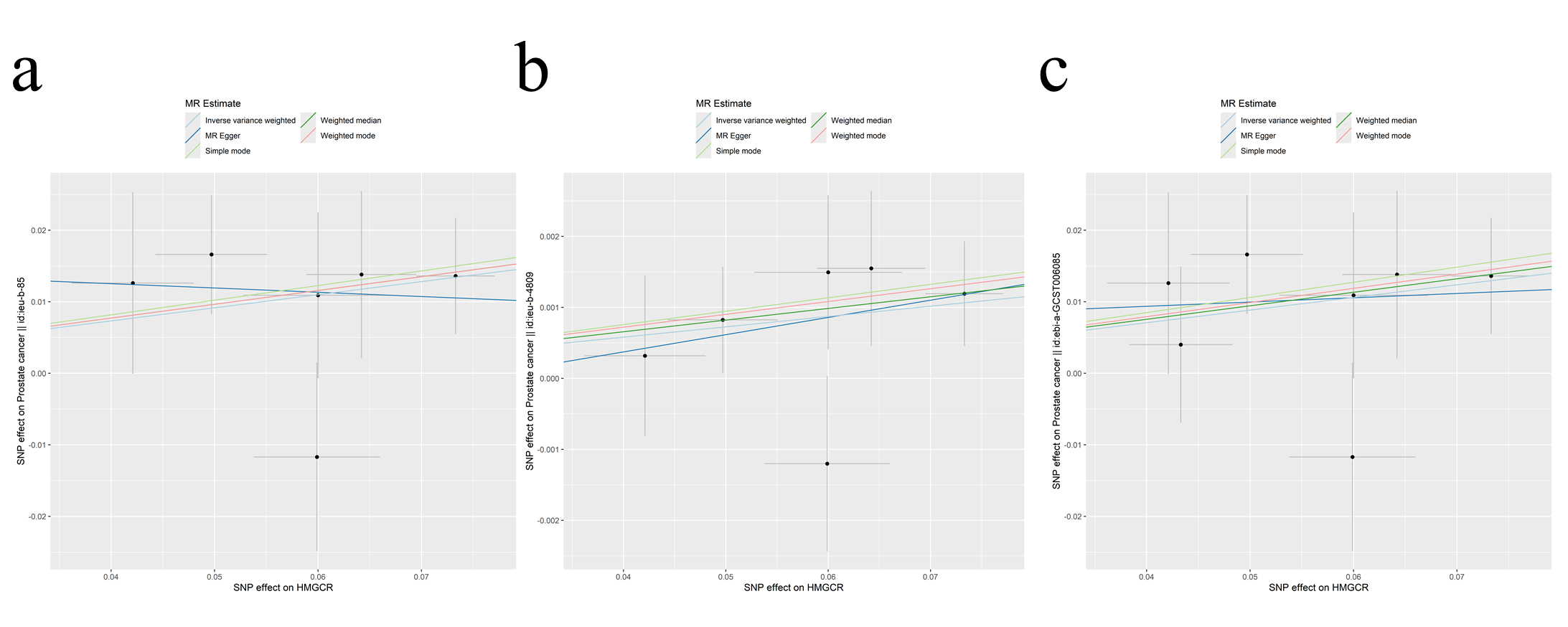


Supplementary-Figure 3. Results of scatter plot: (a) Prostate cancer (GWAS ID: ieu-b-85); (b) Prostate cancer (GWAS ID: ieu-b-4809); (c) Prostate cancer (GWAS ID: ebi-a-GCST006085). GWAS, Genome-Wide association study.


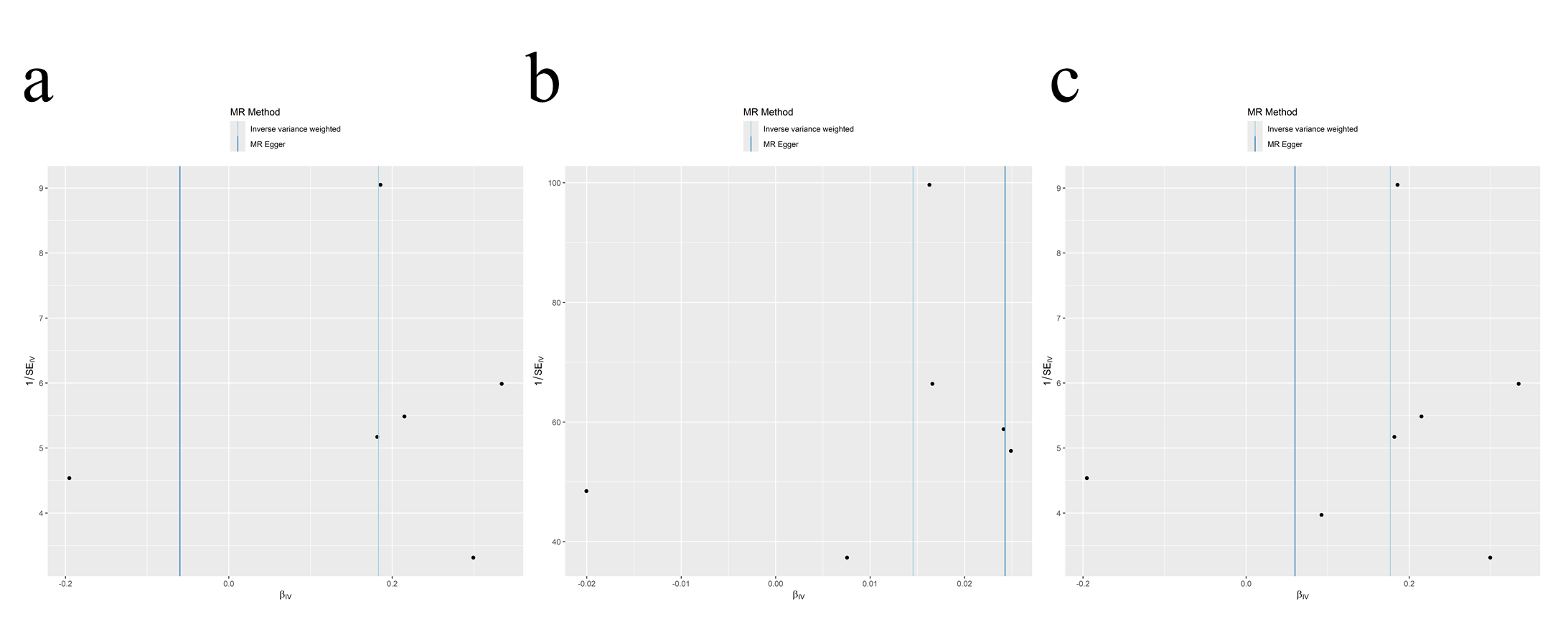


Supplementary-Figure 4. Results of funnel plot: (a) Prostate cancer (GWAS ID: ieu-b-85); (b) Prostate cancer (GWAS ID: ieu-b-4809); (c) Prostate cancer (GWAS ID: ebi-a-GCST006085). GWAS, Genome-Wide association study.
